# Evaluation of rice bacterial blight severity from lab to field with hyperspectral imaging technique

**DOI:** 10.3389/fpls.2022.1037774

**Published:** 2022-10-19

**Authors:** Xiulin Bai, Yujie Zhou, Xuping Feng, Mingzhu Tao, Jinnuo Zhang, Shuiguang Deng, Binggan Lou, Guofeng Yang, Qingguan Wu, Li Yu, Yong Yang, Yong He

**Affiliations:** ^1^ College of Biosystems Engineering and Food Science, Zhejiang University, Hangzhou, China; ^2^ Zhuji Agricultural Technology Extension Center, Zhuji, China; ^3^ College of Computer Science and Technology, Zhejiang University, Hangzhou, China; ^4^ College of Agriculture and Biotechnology, Zhejiang University, Hangzhou, China; ^5^ Agricultural Experiment Station & Agricultural Sci-Tech Park Management Committee, Zhejiang University, Hangzhou, China; ^6^ State Key Laboratory for Managing Biotic and Chemical Treats to the Quality and Safety of Agro-Products, Key Laboratory of Biotechnology for Plant Protection, Ministry of Agriculture, and Rural Affairs, Zhejiang Provincial Key Laboratory of Biotechnology for Plant Protection, Institute of Virology and Biotechnology, Zhejiang Academy of Agricultural Science, Hangzhou, China

**Keywords:** plant disease, hyperspectral imaging, spectral index, deep learning, attention mechanism

## Abstract

Hyperspectral imaging technique combined with machine learning is a powerful tool for the evaluation of disease phenotype in rice disease-resistant breeding. However, the current studies are almost carried out in the lab environment, which is difficult to apply to the field environment. In this paper, we used visible/near-infrared hyperspectral images to analysis the severity of rice bacterial blight (BB) and proposed a novel disease index construction strategy (NDSCI) for field application. A designed long short-term memory network with attention mechanism could evaluate the BB severity robustly, and the attention block could filter important wavelengths. Best results were obtained based on the fusion of important wavelengths and color features with an accuracy of 0.94. Then, NSDCI was constructed based on the important wavelength and color feature related to BB severity. The correlation coefficient of NDSCI extended to the field data reached -0.84, showing good scalability. This work overcomes the limitations of environmental conditions and sheds new light on the rapid measurement of phenotype in disease-resistant breeding.

## 1 Introduction

Rice bacterial blight (BB) is a bacterial vascular bundle disease caused by Xanthomonas oryzae pv. Oryzae (*Xoo*). Since it was discovered in Japan in 1884, the incidence of BB has gradually expanded and spread to major rice-producing countries such as East Asia and Southeast Asia (Elizabeth Sunny and Kumar Shanmugam, 2021). Now BB has spread to various rice-producing areas in the world, such as Africa, America, and Asia, to varying degrees (Fiyaz et al., 2022). BB can occur during the whole growth period of rice and reduce rice production by 10%-20% (Ezuka and Kaku, 2000). The infection is most serious in the late booting stage, which can cause more than 50% yield loss (Kim and Reinke, 2019).

Chemical control is the main measure for rice disease control. Long-term use of chemical agents may lead to drug resistance of pathogens, which will not only fail to control the disease effectively but also cause environmental pollution (Li et al., 2014). In contrast, applying disease-resistant breeding to control BB is an economic and effective alternative method and an important strategy for implementing green disease prevention and control (Martínez-Diz et al., 2019). Disease resistance breeding relies on the mining of BB resistance genes. 44 BB resistance genes have been identified with the development of whole genome sequencing techniques, and 15 of them have been cloned (Yin et al., 2018; Yang et al., 2022). For disease-resistant information mining and identification, accurate measurement of phenotypic information is an essential step. Due to the complexity and dynamic characteristics of plant phenotypes, the traditional evaluation method of disease severity in breeding disease-resistant cultivars has the problems of time-consuming and labor-intensive and uneven data quality (N. Devasena et al., 2018).

Although the study of phenomics is relatively lagging compared to the development of genomics, optical technique, remote sensing, and machine learning methods support the high-throughput collection and intelligent processing of crop phenotypic information (Barbedo, 2019; Bock et al., 2020). Hyperspectral imaging (HSI) technique provides a method to obtain plant phenotypic traits rapidly and non-destructively. The images acquired by HSI contain rich information, which are three-dimensional data cubes composed of hundreds of spectral images. Narrow bands in the visible and near-infrared hyperspectral images are sensitive to the subtle changes of plants caused by diseases, providing the possibility for disease identification and diagnosis (Yuan et al., 2019; Lin et al., 2020). Screening important bands related to target tasks as features (such as calculating vegetation index) is effective to reduce redundant data in hyperspectral bands (Marin et al., 2021; Zhang et al., 2021). There are also related researches on constructing new indices for disease detection by screening sensitive bands. Abdulridha et al. (2020b) tested 29 spectral vegetation indices and determined the most suitable indices for distinguishing different developmental stages of powdery mildew in squash. Zhang et al. (2018a) found three important bands for the identification of the damage severity of pine by different waveband selection algorithms. Moreover, texture, color, shape and other features in the image provide diverse and useful information (Lu et al., 2018; Smigaj et al., 2019). Guo et al. (2020) have improved the evaluation accuracy of wheat leaf yellow rust with different damage severity by 6.3% by using the fusion of six characteristic wavelengths of hyperspectral images and the preferred four texture features. It can be seen that multi-feature fusion provides a new idea for evaluating crop disease, and extracting the feature related to the target in the hyperspectral images is the key to the process of hyperspectral data.

Establishing effective analytical models for data interpretation is a significant part of phenotyping based on hyperspectral imaging. Deep learning methods have been applied to the phenotypic analysis of crop diseases in lab and field environments with the advantages of powerful automatic learning and feature extraction (Zhang et al., 2018b; Too et al., 2019; Prabhakar et al., 2020). Duarte-Carvajalino et al. (2018) found that convolutional neural network (CNN) network was better than multilayer perceptron and support vector machine (SVM) in predicting the severity of potato late blight. Geetharamani and Arun Pandian (2019) proposed a nine-layer deep convolutional neural network model to identify plant leaf diseases. They acquired the prediction accuracy was 9.78% higher than that of the K-nearest neighbors model. Compared with conventional methods, deep learning methods have better performance and advantages (Zhang et al., 2019).

In addition, deep learning methods have shown good capabilities in feature selection. Garg and Alam (2022) proposed a method combining a pre-trained CNN network and long short-term memory (LSTM) network, which made the proposed model more focused on finding target-related information in the input data and improved the classification results of apple leaf diseases. Mi et al. (2020) embedded the convolution block attention module in the CNN to make the network pay more attention to the key areas in making the decision and realized the classification of wheat stripe rust in a lab environment with an accuracy of 97.99%. Almost all studies were conducted in a lab environment and based on a single period to evaluate the disease. However, crop diseases become severe over time. It is significant to grasp the dynamic development of disease severity for disease control and disease-resistant breeding. Moreover, most existing research results were obtained in a controlled environment. In fact, crops grow in a complex field environment. The data acquired in the field are affected by various factors such as soil, water, light, etc. We will focus on that whether the results obtained in the lab environments can be extended to complex environments.

**Figure 1:**
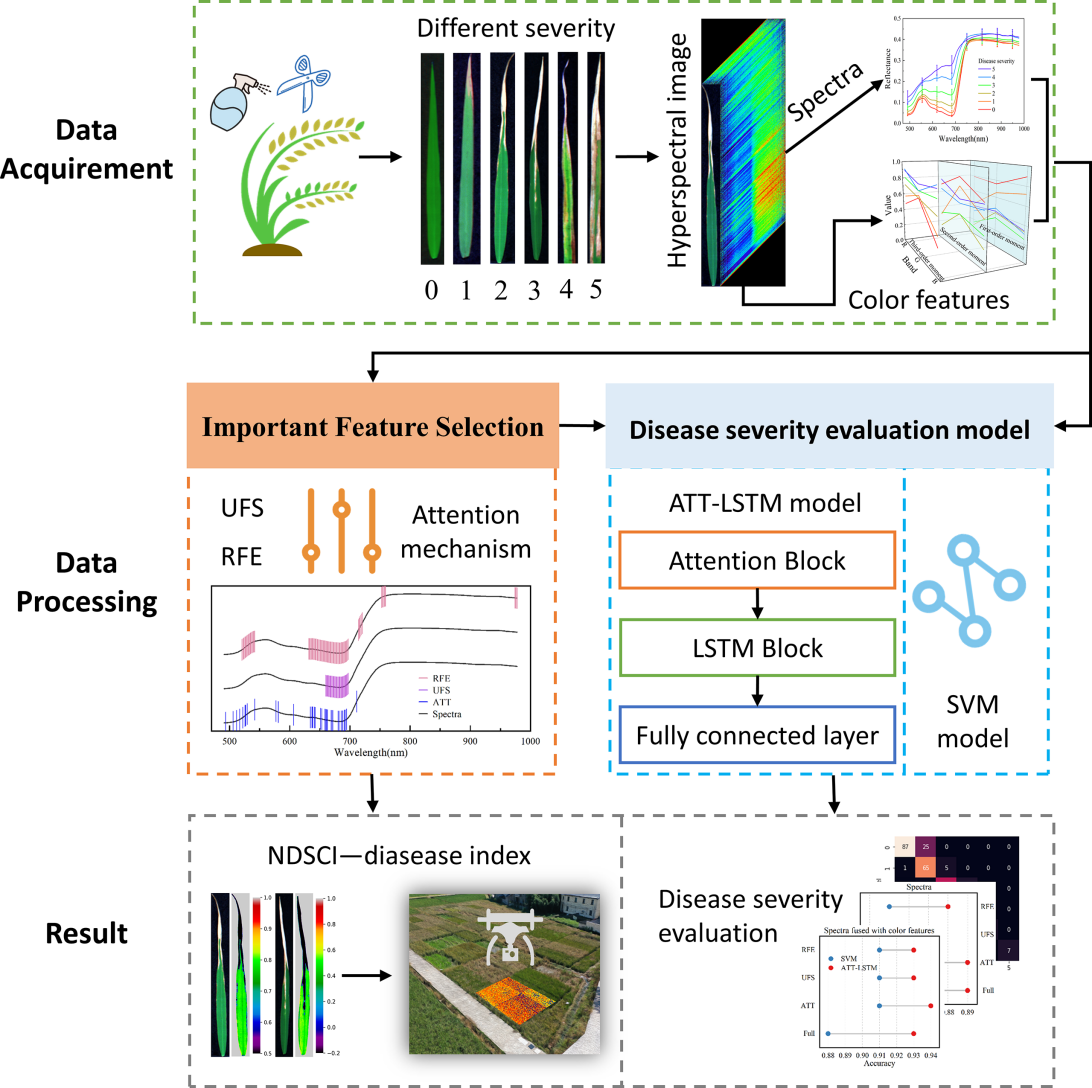
Workflow of the study.

This study aims to explore the development of rice BB severity based on time-series hyperspectral images and deep learning methods and achieve an accurate evaluation of disease severity. The specific content is as follows: (1) screening out important information from a large amount of raw data; (2) establishing a robust and accurate BB severity evaluation model; (3) proposing a disease index construction strategy to achieve BB severity evaluation from the lab to the field. **Figure 1** shows the workflow of the study.

## 2 Material and methods

### 2.1 Plant materials

Three rice cultivars, IR24, 3A26, and 4A37, were used in this study. IR24 is susceptible to BB. 3A26 and 4A37 are resistant to BB by introducing the quantitative trait loci (QTL) related to the resistant ability into IR24. The resistant QTL of 3A26 is mapped on chromosome 5, and the resistant QTLs of 4A37 are mapped on chromosome 5 and chromosome 3. Their resistance to BB has been reported in the study by Han et al. (2014). The rice samples were provided by State Key Laboratory Breeding Base for Zhejiang Sustainable Pest and Disease Control, Hangzhou, China. The samples were planted in the experimental field at Zhejiang Academy of Agricultural Science, Hangzhou, China. The size of the planting plot was 2m×15m with a row spacing of 50cm. Growth management followed normal agricultural practices.

On September 6, 2021, rice was inoculated with pathogens at the beginning of the booting stage (approximately 40 days after transplanting). An artificial leaf tip removal method was used to inoculate rice with BB. Using a scissor which should be dipped in the solution of *Xoo* with the optical density of 0.8, cut off the tip tissues of the fully expanded rice leaves about 3 mm. Then, the solution was poured into a watering can for spraying to ensure that all the rice in the infected group could be fully covered by the solution droplets. At the same time, the healthy control group was cut by a scissor dipped in pure water and sprayed with pure water. The samples were collected on the 3^rd^, the 13^rd^, the 33^rd^, and the 39^th^ days after inoculation according to the development of BB lesions. The complete inoculated full expanded leaves were selected as experimental samples for subsequent acquisition of hyperspectral images and physiological parameters. A total of 450 samples were collected. The specific sample collection is detailed in **Table S1**.

### 2.2 Hyperspectral image acquisition and spectra extraction

A visible/near-infrared (VIS-NIR) hyperspectral imaging system was used to acquire hyperspectral images of rice leaves. The spectral range is 414-1017nm with 473 bands. The whole system consists of an imaging spectrometer (ImSpector V10E; Spectral Imaging Ltd., Oulu, Finland), a highly sensitive EMCCD camera (Raptor EM285CL, Raptor Photonics limited, Larne, United Kingdom) with a long camera lens (OLES23; Specim, Spectral Imaging Ltd., Oulu, Finland), two 150 W tungsten halogen lamps (3900 Lightsource, Illumination Technologies Inc., United States), and a conveyor belt driven by a stepper motor (Isuzu Optics Corp., Taiwan, China). The two 150 W tungsten halogen lamps are placed symmetrically on both sides of the lens as the illumination. The system is controlled by a computer through the Spectral Image-V10E software (Isuzu Optics Corp., Taiwan, China).

The distance between the camera lens and samples was set to 29 cm, the exposure time of the camera and the intensity of the illumination module were set to 45 ms and 180, and the speed of the conveyor belt was set to 2.2 mm/s. The acquired hyperspectral images of samples should be corrected to be analyzed. The correction method and formula were consistent with the previous study (Bai et al., 2020). Then, the whole leaf was defined as the region of interest (ROI), and the hyperspectral image at 792 nm was selected to build a mask to remove background information with a threshold value of 0.1. Each pixel of the ROI could be extracted. Wavelet transform (Daubechies: 10; Decomposition level: 3) was used to de-noise the extracted pixel-wise spectra. The average spectra of each ROI were calculated as the corresponding sample spectrum. The extracted spectra were processed by baseline correction to prevent errors caused by different acquisition times. The spectra in the 490-978 nm (380 bands) were used for analysis due to obvious noise in the head and tail spectra.

### 2.3 Disease severity definition

The threshold segmentation method was used to calculate the area of the healthy and diseased parts. In section 2.2, the ROI hyperspectral image without background information could be acquired. Experts manually marked the lesion parts in the ROIs by utilizing software ENVI 4.7 ((ITT Visual Information Solutions, Boulder, CO, USA). The pixel summation of the infected area and the whole rice leaf area were obtained, and the ratio of the infected area over the whole rice leaf area could be calculated according to the following equation:


$$R_{l}=\frac{\sum S_{leision}}{\sum S_{WL}}\times 100\%$$

where R_l_ represents the ratio of the infected area and the whole rice leaf area, ∑*S*_*leision*_ and ∑*S*_*WL*_ represent the pixel summation of the infected area and the whole rice leaf area, respectively. A scale of 0-5 was used to measure the disease severity, as shown in **Table 1**. 0 indicated no infection of leaves.

**Table 1:** The evaluation criteria of disease severity.

Disease severity	R_l_
0	0
1	0~10%
2	10%~20%
3	20%~50%
4	50%~75%
5	>75%

R_l_ represents the ratio of the infected area and the whole rice leaf area.

### 2.4 Color feature extraction

The distribution information of color is mainly concentrated in the low-order moments, which are the first-order moment (mean), second-order moment (var), and third-order moment (ske) (Markus Andreas and Markus, 1995). The first-order moment represents the mean response intensity of the color channel, the second-order moment is the response variance of the color channel, and the third-order moment denotes the skewness of the data distribution. The calculation formula could be found in Jiang et al. (2020). RGB values of each pixel in the hyperspectral image of each sample were output (R channel at 657 nm, G channel at 552 nm, and B channel at 450 nm), and a total of 9 color features were extracted.

### 2.5 Data analysis methods

#### 2.5.1 Statistical methods

The differences of spectral data and color features were analyzed by one-way analysis of variance (ANOVA) at a significance level of 0.05. The p-value shows the difference between the data, and the smaller the p-value, the greater the difference (Mokarram et al., 2012). Correlation analysis was used to analyze the correlation between color features and disease severity, and the absolute value of correlation coefficient (r) close to 1 indicated a strong correlation (Xiao et al., 2016).

#### 2.5.2 Conventional machine learning methods

Univariate feature selection (UFS) and recursive feature elimination (RFE) are used for feature extraction. UFS screens important variables based on statistical tests of each variable (Verma and Pal, 2020). The Chi-square test was used to select important wavelengths in this paper. RFE uses a machine learning model to screen important features through multiple training rounds recursively. We use RFE based on SVM to select important wavelengths. SVM-RFE is commonly used, and its detailed formula can be found in (Yan and Zhang, 2015).

SVM is a widely used pattern recognition method and is also used for evaluating disease severity in this study. The SVM model was established using spectral data and color features as input. The radial basis function was used, and the optimal performance is determined by the penalty coefficient (c) and the kernel function parameter (g) (Chandra and Bedi, 2021). The optimal c and g were determined by grid search in 10^-8^ to 10^8^. UFS, RFE and SVM are all implemented based on scikit-learn (https://scikit-learn.org/stable/).

#### 2.5.3 Deep learning method

**Figure 2:**
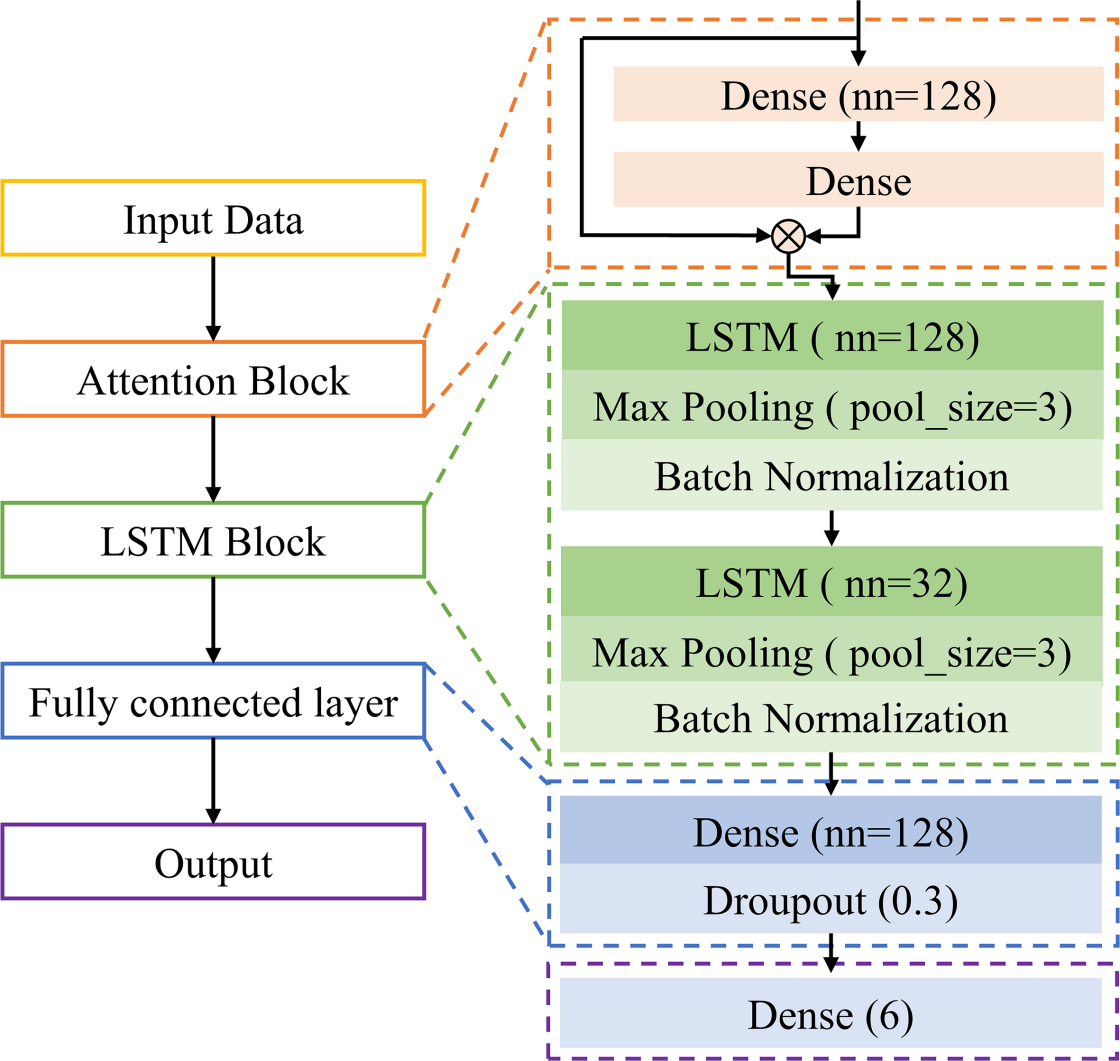
The architecture of ATT-LSTM model.

A self-built long short-term memory network with an attention mechanism (ATT-LSTM) was used to evaluate the disease severity. The architecture of the designed ATT-LSTM model is shown in **Figure 2**.

The first block was ATT block, which was realized by two dense layers:


$$Y_{ATT}=f_{relu}\left(W_{2}⊗f_{relu}(W_{1}⊗\text{X}+b_{1}\right)+b_{2})⊗\text{X}$$

where *Y_ATT_* is the output of the ATT block, *X* denotes the input data, *W* and *b* represent the weights and bias of the attention layer, ⊗ indicates matrix multiplication, and *f_relu_* is the activation function. The number of neurons of the first dense layer was set to 128, and that of the second dense layer was set as the number of input variables. The attention mechanism helps the model assign different weights to each input variable, which makes the network pay more attention to important information (Li et al., 2020). Thus, the ATT block could also be used to extract the feature variables. Define the value of *f*_*relu*_(*W*_2_⊗*f*_*relu*_(*W*_1_⊗*X*+*b*_1_)+*b*_2_) as the ATT weight. Variables with larger weight values are more important. In this study, the important wavelengths were screened based on the ATT weight.

The second part was the LSTM block. It contained two LSTM units with 128 and 32 hidden units, respectively. Each LSTM unit was followed by a max pooling layer and a batch normalization layer. LSTM is a special recurrent neural network that stores long-term states by adding a memory neuron. The neuron can decide which states are forgotten or retained, thus solving the recurrent neural network gradient disappearance or gradient explosion (Zhelev and Avresky, 2019). It has advantages for the processing of time-series data. The specific introduction of LSTM can be seen in http://colah.github.io/posts/2015-08-Understanding-LSTMs/.

The LSTM block was followed by a fully connected layer containing 128 neurons, and a dropout layer was used to prevent overfitting. Finally, output category. The output of this study was the disease severity with 6 categories.

The deep learning model was implemented based on the MXNET framework. The Softmax cross-entropy loss function and adaptive moment estimation were applied to train the model. In the training phase, the batch size was set as 20, and a dynamic learning rate was used. In the beginning, a relatively large learning rate of 0.001 was set to speed up the training process for the first 500 epochs, and then it was reduced to 0.0001 for the next 300 epochs.

#### 2.5.4 Model evaluation

The disease severity evaluation model was established based on spectral data, color features, and the fusion of spectral data and color features. The input data was divided into calibration set and prediction set at a ratio of 7:3 to train the model. To quantitatively evaluate the performance of the model, the accuracy of different severity (ACC.), precision (Pre.), recall (Rec.), and F1-score (F1.) were calculated. The calculation formulas were referred (Vujovic, 2021).

### 2.6 New index for disease severity evaluation

A disease index—that could reflect the disease severity based on important wavelengths and color features was constructed. The strategy for constructing the new spectral index is to rely on the combination of the most important wavelength through the disease degree evaluation model and the color feature with the highest correlation with disease severity. Inspired by the idea of normalized difference vegetation index, the normalized difference spectral color index (NDSCI) was established, and the formula is as follows:


$$NDSCI=\frac{H_{c}-R_{i}}{H_{c}+R_{i}}$$

where H_c_ is the color feature most related to disease severity in hyperspectral images and R_i_ is the reflectance of the most important wavelength selected from spectral data.

The selected color feature and spectral reflectance values were normalized to the range of [0,1] for displaying the variation of NDSCI intuitively. For the NDSCI, normalized difference vegetation index (NDVI), renormalized difference vegetation Index (RDVI), red-edge vegetation stress index (RVSI), red-edge chlorophyll index (CI_red-edge_), green chlorophyll index (CIgreen), MERIS terrestrial chlorophyll index (MTCI), Green Normalized Different Vegetation Index (GNDVI), different spectral index (DSI), optimized soil-adjusted vegetation index (OSAVI), photochemical reflectance index (PRI), enhanced vegetation index (EVI), and water index (WI) were compared by correlation analysis with the disease severity. The formulas for calculating the spectral indices are shown in **Table S2**. The wavelength of 682nm and the first moment of the B channel (B_mean_) were used to construct the NDSCI in this study. The results are described in section 3.4.

## 3 Results

### 3.1 Spectral profile and color features analysis

**Figure 3:**
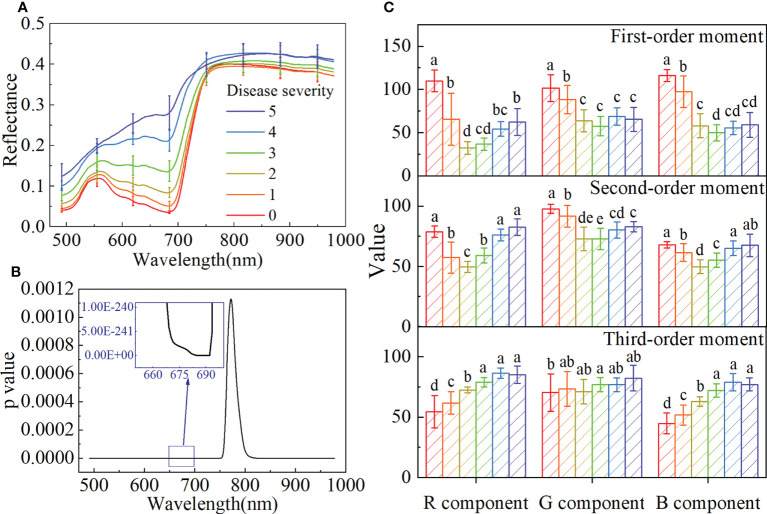
The spectral features and color features of the samples with different disease severity. **(A)** The average spectra with standard deviation. **(B)** The p value obtained by ANOVA for the spectra with different disease severity. **(C)** Bar charts with error bars of color features and different letters indicate significant differences between different disease severity using the Tukey method (P<0.05).

**Figure 3A** shows the average spectra with a standard deviation of different disease severity. With the seriousness of BB, the spectral reflectance gradually increased in the visible range of 480-780 nm, and the spectra were significantly different in the near-infrared (NIR) spectral range of 780-980 nm. The spectral differences between the healthy and infected rice were analyzed by ANOVA. As shown in **Figure 3B**, p values are less than 0.05 at all wavelengths, and the minimum value appears in the 680-690 nm range. It indicated significant differences between the spectra of the different disease severity.

The color features of different disease severity are shown in **Figure 3C** and **Table S3**. With the seriousness of BB, the first-order moment of the B component gradually decreased, the third-order moment of the R and B components gradually increased, and the second-order moment of all color components showed a trend of first decreasing and then increasing. Although the trends of these color components were different, it could be proved that there were differences among different disease severity.

The changes and differences in spectral and color features of rice with different BB severity indicated that it is feasible to use these to evaluate the disease severity.

### 3.2 Disease severity evaluation based on full spectra and color features

Spectral data and color features were inputted to ATT-LSTM and SVM models. **Table 2** and **Table S4**-**1** show the statistical results of the prediction and calibration sets, respectively. In contrast, no matter which model was used, the accuracy of the evaluation of disease severity based on spectral data was higher than that of analysis based on color features. This might be due to the spectral data containing more information than color features, which was more conducive to evaluating disease severity. The ATT-LSTM model based on spectral data achieved satisfactory results, with an accuracy of 0.89.

**Table 2:** The prediction results of ATT-LSTM and SVM models by using spectral data and color features.

Model	Class^a^	Spectral data				Class	Color features			
				Pre.	Rec.		F1.	Acc.		Pre.	Rec.	F1.	Acc.
ATT-LSTM	0	0.98	0.98	0.98	0.89	0	0.93	0.95	0.94	0.83
	1	0.91	0.98	0.94		1	0.9	0.86	0.88	
	2	0.89	0.80	0.84		2	0.76	0.81	0.79	
	3	0.92	0.92	0.92		3	0.88	0.88	0.88	
	4	1.00	0.67	0.80		4	0.67	0.50	0.57	
	5	1.00	1.00	1.00		5	0.75	1.00	0.86	
SVM	0	0.96	0.98	0.97	0.86	0	0.86	0.97	0.92	0.83
	1	0.91	0.93	0.92		1	0.92	0.79	0.85	
	2	0.82	0.70	0.76		2	0.63	0.75	0.69	
	3	0.85	0.92	0.88		3	0.81	0.71	0.76	
	4	1.00	0.67	0.80		4	0.60	0.75	0.67	
	5	1.00	1.00	1.00		5	1.00	1.00	1.00	

Acc., Accuracy; Pre., Precision; Rec., Recall; F1., F1-score.

a Class represents the label of the disease severity.

In addition, the fusion of spectral data and color features was considered. **Table 3** shows the prediction results of ATT-LSTM and SVM models, and **Table S4**-**2** shows the calibration results. Compared with **Table 2**, better results could be obtained after the data fusion. The prediction results of the ATT-LSTM model and the SVM model after data fusion were 4.49% and 1.16% higher than those using spectral data, respectively. Correspondingly, for color features, the prediction results were increased by 12.48% and 4.82%, respectively. Data fusion could effectively improve the evaluation effect of disease severity.

**Table 3:** The prediction results of ATT-LSTM and SVM models by fusing spectral data and color features.

Class^a^	ATT-LSTM				Class	SVM			
		Pre.	Rec.	F1.		Acc.		Pre.	Rec.	F1.	Acc.
0	0.95	0.95	0.95	0.93	0	0.90	0.95	0.92	0.87
1	0.95	0.93	0.94		1	0.92	0.86	0.89	
2	0.81	0.81	0.81		2	0.80	0.75	0.77	
3	0.88	0.92	0.90		3	0.85	0.96	0.90	
4	1.00	1.00	1.00		4	1.00	0.75	0.86	
5	1.00	1.00	1.00		5	1.00	1.00	1.00	

Acc., Accuracy; Pre., Precision; Rec., Recall; F1., F1-score.

a Class represents the label of the disease severity.

Although both ATT-LSTM and SVM models exhibit good performance, the ATT-LSTM model obtained better results. By comparison, the results of the ATT-LSTM model were better than that of the SVM model when the disease severity was less than 4 (**Table 2**). For example, when the disease severity of 2, the Pre., Rec., and F1. of the ATT-LSTM model based on the spectral data were 0.89, 0.80, and 0.84, respectively, while those of the SVM model were 0.82, 0.76 and 0.76, respectively. Similarly, the values of Pre., Rec., and F1 of the SVM model were lower than those of the ATT-LSTM model when modeling with color features. ATT-LSTM model was better at subtle mining features, which benefited the discrimination of the mild disease severity. In the case of data fusion, the accuracy of the ATT-LSTM model was 6.90% higher than that of the SVM model, and the values of Pre., Rec., and F1. under different disease severity were higher than that of the SVM model (**Table 3**).

### 3.3 Disease severity evaluation based on feature extraction

**Figure 4:**
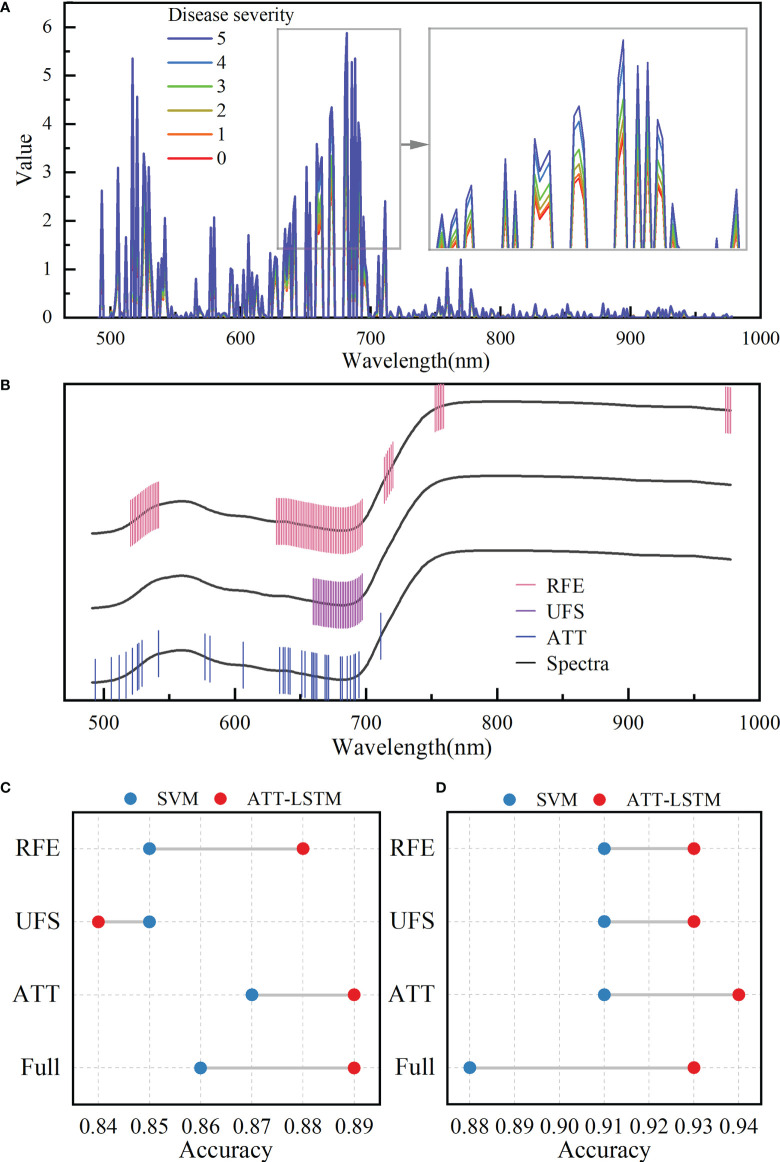
Important wavelength selection results. **(A)** Attention weights of different disease severity calculated by the attention block. **(B)** Important wavelengths selected by different methods. The black lines represent spectral profiles and are used only to visualize the selected wavelengths. The results of disease severity evaluation models were established by using the selected important wavelengths **(C)** and important wavelengths fused with color features **(D)** (RFE, UFS, and ATT represent for modelling with the extracted important wavelengths using the corresponding feature extraction methods, respectively; Full represents for modelling with all wavelengths).

There was a correlation between spectral bands, as illustrated in **Figure S1**. Extracting useful information could reduce the burden of the model with less information redundancy. The values of attention weight of each band based on the ATT block in the ATT-LSTM model are shown in **Figure 4A**. There were differences in the ATT weight values among different disease severity. Wavelengths with the weight value greater than 1 were selected as important features for subsequent analysis. **Figure 4B** shows the locations of important wavelengths selected by ATT, UFS, and RFE methods. The UFS method selected 30 important wavelengths, mainly in the range of 660-698nm. The important wavelengths found by the RFE method were distributed in 521-542nm, 632-697nm, 714-720nm, 753-759nm, and 974-978nm, with a total of 86 important wavelengths. The 34 important wavelengths selected by the ATT method were relatively uniformly distributed in the visible range. The important wavelengths selected by the abovementioned methods were highly coincident in the 634-692nm range, indicating that this range was extremely important.

ATT-LSTM and SVM models were established based on the important wavelengths selected by different feature extraction methods. **Figure 4C** and **Table S5** show the accuracy of the prediction set and the results of the calibration set, respectively. The important wavelengths selected by ATT methods could achieve the highest prediction accuracy regardless of the modeling method. It even surpassed the results based on the full spectra for the SVM model. It proved the validity of the ATT feature extraction method. For the ATT-LSTM model, the lowest accuracy was obtained using the UFS method. This could be due to the screened important wavelengths being clustered in a region containing limited effective information. In addition, the accuracies of the ATT-LSTM models established on different feature extraction methods were higher than those of SVM models, except for the UFS method. It also showed the proposed ATT-LSTM model was suitable for evaluating the BB severity.

The important wavelengths selected by different methods were fused with color features to evaluate the disease severity, and the results are shown in **Figure 4D** and **Table S6**. The ATT-LSTM model based on the wavelengths selected by the ATT method realized the highest prediction accuracy of 0.94, which was higher than that of the model based on full spectra. Moreover, the prediction accuracies of the different models were improved after data fusion compared with **Figure 4C**. Effective wavelength selection and data fusion were positive in evaluating the disease severity.

### 3.4 NDSCI index

**Figure 5:**
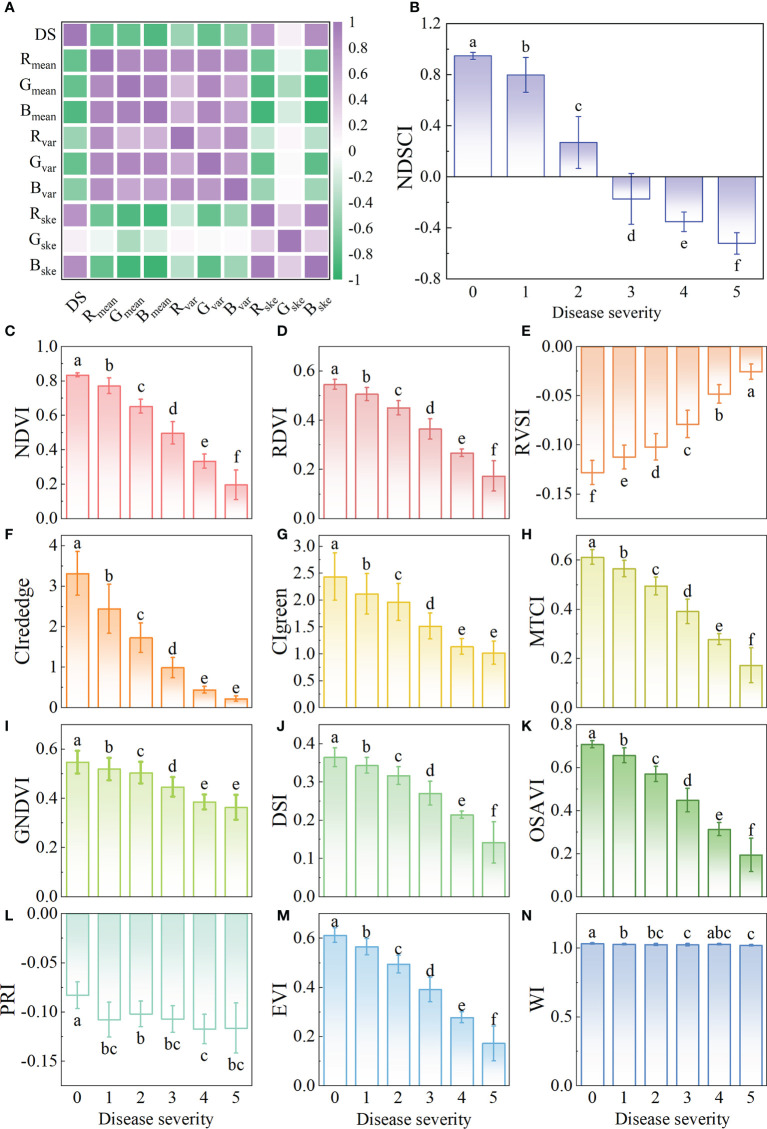
Results of the construction of new spectral index. **(A)** The correlation of the color features and disease severity. The variation of **(B)** NDSCI, **(C)** NDVI, **(D)** RDVI, **(E)** RVSI, **(F)** CI_rededge_, **(G)** CIgreen, **(H)** MTCI, **(I)** GNDVI, **(G)** DSI, **(K)** OSAVI, **(L)** PRI, **(M)** EVI, and **(N)** WI. Different letters indicate significant differences between different disease severity using the Tukey method (P<0.05). Black plots represent the lodging of rice in the plot, which are not considered in the experiment.

From the foregoing results, the wavelength of 682nm was the important spectral feature. In addition, B_mean_ had the highest correlation with disease severity, with a correlation coefficient of -0.84 (**Figure 5A** and **Table S7**), which was the important color feature. NDSCI was constructed based on the important wavelength and color feature. The variation of the NDSCI is shown in **Figure 5B**. It could be observed that the value of NDSCI continuously decreased with the seriousness of BB. The correlation coefficient between the NDSCI and disease severity was -0.93. The value of NDSCI was less than 0 when the disease severity exceeded 2. In this way, the critical point of disease severity could be distinguished.

**Figure 6:**
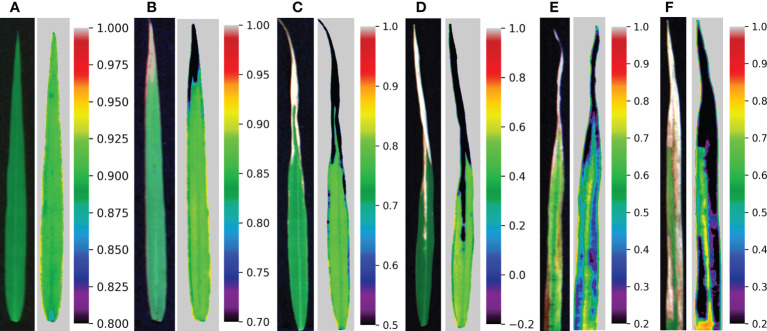
RGB images and corresponding visualization images of NDSCI. From left to right are disease severity of **(A)** 0, **(B)** 1, **(C)** 2, **(D)** 3, **(E)** 4, and **(F)** 5, respectively.

The correlation between common spectral indices and disease severity is shown in **Table 4**, and the variations of these indices are shown in **Figures 5C–N**. The spectral indices have a good correlation with disease severity except for PRI and WI, among which the correlation coefficient of NDVI and CI_red-edge_ reached -0.91. However, there was a certain overlap between the values of NDVI and CI_red-edge_ among different disease severity by comparing **Figures 5B, C, F**. For the value of NDSCI, the differences in the disease severity were obvious. Especially when the disease severity was greater than 2, the value of NDSCI was less than 0, which provided the feasibility of evaluating the disease severity rapidly. **Figure 6** shows the pseudo-color images calculated by NDSCI. Compared with RGB images, the difference between the lesion area and healthy area was digitalized and displayed on the NDSCI visualization images. It could be obviously observed that the NDSCI value of the healthy area was significantly higher than that of the lesion area, and the lesion areas were highlighted. Moreover, the area of the disease-health junction that could not be easily observed through the RGB image could be visually displayed on NDSCI visualization images. The NDSCI value of the disease-health junction was between the value of the healthy area and the lesion area, which showed the changes of the disease vividly.

**Table 4:** Correlation coefficient (r) values between different spectral indices and disease severity.

Spectral index	r value	Spectral index	r value
**NDSCI**	**-0.93**	GNDVI	-0.79
NDVI	-0.91	DSI	-0.62
RDVI	-0.81	OSAVI	-0.84
RVSI	0.73	PRI	-0.37
CIred-edge	-0.91	EVI	-0.77
CIgreen	-0.82	WI	-0.24
MTCI	-0.77		

**Figure 7:**
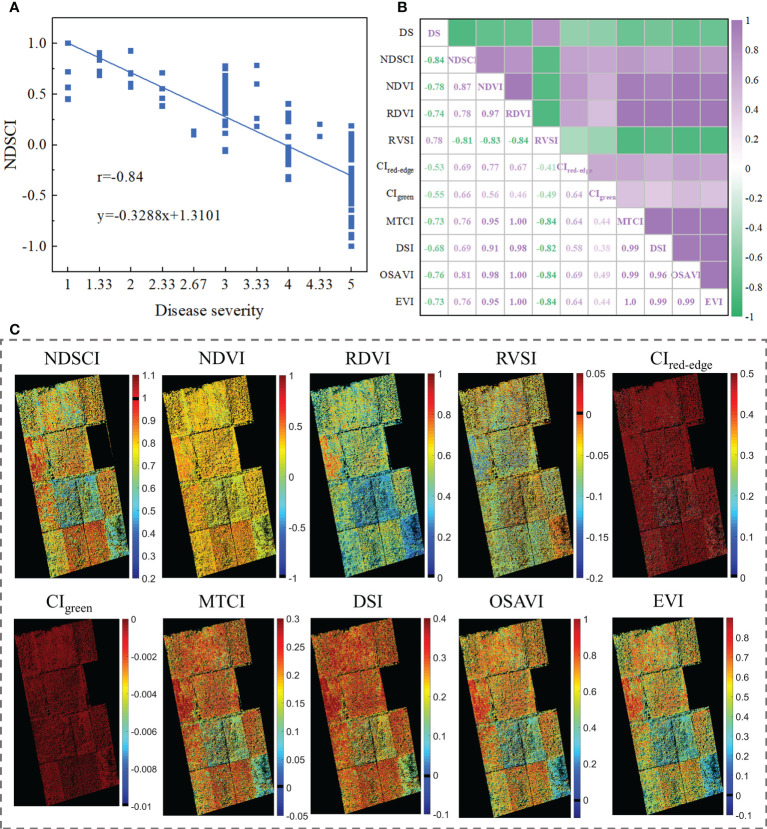
Results of NDSCI extension to field data. **(A)** Scatter plot of the NDSCI of the field. **(B)** The correlation of the indices and disease severity. **(C)** Disease severity maps calculated by NDSCI, NDVI, RDVI, RVSI, CI_red-edge_, CIgreen, MTCI, DSI, OSAVI, and EVI.

To test the scalability of NDSCI, images of rice fields stressed by *Xoo* collected by an unmanned aerial vehicle platform on September 26, 2021, were used for validation. Detailed information is shown in **Figure S2** and **Table S8**. The acquired RGB images were converted to HSV space to reduce the impact of changes in lighting conditions. Based on the NDSCI strategy, we found that the most important wavelength was 675nm and the color feature was the first moment of the S channel. There was a correlation between the finally established NDSCI and the disease severity, and the r reached -0.84, as shown in **Figure 7A**. **Figure 7B** shows the correlations between some commonly used indices and disease severity. NDSCI obtained the highest correlation with the disease severity, showing the feasibility and scalability of its application in the field. In addition, the distribution of disease severity in the field was visualized using NDSCI and vegetation indices, as presented in **Figure 7C**. The scores of CI_red-edge_ and CI_green_ were close among different plots, and the two spectral indices were not suitable for evaluating the severity of BB. For NDVI, RDVI, RVSI, MTCI, DSI, OSAVI and EVI, the scores of different plots were different, but the plots with similar disease severity could not be well distinguished. In contrast, NDSCI values could clearly show the disease severity development in the field.

## 4 Discussion

### 4.1 Attention mechanism is suitable for feature selection

Compared with conventional machine learning methods, deep learning methods have advantages in feature learning and classification tasks. When using the self-built ATT-LSTM model to evaluate the disease severity, the weight value obtained by the ATT block showed that the weight value of each wavelength was different under different disease severity (**Figure 4A**). This was different from the results obtained by conventional feature selection methods. For example, UFS is an unsupervised feature selector which performs variable selection based on a chi-square test between each feature and has nothing to do with the output. According to **Figure 4B**, it could be found that the important wavelengths selected were gathered in the parts with large differences between different spectra. The valid information identified by UFS was limited. For the RFE method, the results depend on the selection of the base model, and the number of features to retain needs to be specified before filtering. However, how many features will be valid is generally unknown. The severity of rice was constantly changing after BB infection, and different samples have their characteristics. The different weight values of ATT demonstrated the superiority of deep learning methods. In this study, the attention mechanism made the model more focused on the important features, and the LSTM model could capture and process the information well in the time-series data. The ATT-LSTM model performed better than the SVM model. Compared with Zhang et al. (2022)’s study on rice BB severity evaluation based on attention mechanism and convolutional neural network model, the accuracy of this study increased by 4.82%. The proposed ATT-LSTM is suitable for evaluating rice BB severity, and the attention block is conducive to feature selection.

### 4.2 Data fusion improves the results of disease severity evaluation

In the research of crop diseases, it is feasible to detect the disease based on spectral data or features of images (Muhammed, 2005; Yuan et al., 2019; Zhang et al., 2020). Better results in the evaluation of rice BB severity can be obtained by the data fusion of spectra and color features in this study.

The evaluation of disease severity using spectral data and color features could obtain acceptable results. It was noted that both SVM and ATT-LSTM models acquired better results than before after using the fused data (**Table 2**). The fusion of spectral and color features provided information about the disease’s development. Extracting the information on disease development from multiple perspectives could fully reveal the development of disease. In addition, the hyperspectral data contained 380 bands, and the correlation analysis showed a strong correlation between the adjacent bands (**Figure S1**). After feature wavelength selection by the ATT block in the proposed ATT-LSTM model, the evaluation effect of the disease severity was improved, indicating that there was data redundancy in the raw hyperspectral data. The model’s performance improved further when the selected wavelengths and color features were fused. The fusion of effective data could achieve satisfactory results. In this study, only the spectral data and color features were simply the primary fused of the raw data. Feature-level and decision-level data fusion can be explored in the subsequent analysis.

### 4.3 NDSCI can be used to evaluate BB severity from lab to field

Guided by the ATT mechanism and data fusion strategy, the NDSCI index was obtained. The ATT weight values indicated the highest score at the wavelength of 682nm, where the ANOVA also showed significant differences between the spectra of different severity (**Figure 3B**). Currently, the extraction of information from hyperspectral images is mainly based on the differences of different bands or through statistical methods to select target-sensitive bands. In fact, the color change of rice under BB stress could also be reflected in hyperspectral images. Color moments are simple and effective representations of color features. In this study, the color features of samples with different disease severity were different in the RGB color space, which was consistent with the result of Sabrol and Kumar (2016). B_mean_ was selected, which was the mean response intensity of the B channel. The B channel is sensitive to crop disease infection and can reflect the color change of rice under *Xoo* stress. Satisfactory results were obtained by combining the important wavelength and color feature to construct the new disease index.

High-quality hyperspectral data can be obtained in the lab environment, while the quality of data obtained in the field environment is affected by changes in environmental conditions such as sunlight intensity and wind. Differences in data quality make it difficult for results to cross from lab to field. The important features we got in the lab setting were not the same as in the field, which was the same as the result of Abdulridha et al. (2020a). The important features varied with the environmental conditions change, but these features were closely related to BB severity. The strategy of constructing NDSCI relied on the important features of the acquired data to associate with the target task, which overcame the phenomenon of differences in the lab and field environments. Few studies have explored the extension from the lab environment to the field environment. It is commendable that research based on the lab environment can be truly extended to various applications. The current research is a preliminary attempt, and it is necessary to explore the principle in the future further.

## 5 Conclusion

In this study, time-series VIS-NIR hyperspectral images collected from different resistant rice cultivars infected with BB were analyzed for the evaluation of the disease severity. The results indicated that spectral data and color features were reliable for evaluating rice BB severity, and the data fusion could further improve the evaluation results. The idea of data fusion provided a new idea for the precise measurement of plant phenotypes. Moreover, compared with conventional machine learning methods, the proposed ATT-LSTM model showed better performance. Deep learning provided a reliable method for the evaluation of rice BB severity and the selection of important features. Inspired by satisfactory results that could be obtained by the fusion of spectra data and color features, NDSCI was constructed. NDSCI showed the advantages of evaluating the BB severity compared with commonly used spectral indices. The NDSCI strategy realized the leap from the lab to the field, providing a basis for future related research. Timely and effectively controlling the occurrence and development of BB is a benefit for the growth and production of rice. This study could provide a reliable method for understanding the development of rice BB, and provide assistance for breeders to obtain the disease extent.

## Data availability statement

The original contributions presented in the study are included in the article/**Supplementary Material**. Further inquiries can be directed to the corresponding author.

## Author contributions

Conceptualization, XB and XF; methodology, XB, MT, JZ, GY, and QW; software, XB; validation, XB, SD, and BL; formal analysis, XB and XF; investigation, XB, YH, and YZ; resources, XF and YH; data curation, YY and XF; writing—original draft preparation, XB; writing—review and editing, YH and XF; visualization, XB; supervision, XF and LY; funding acquisition, XF and YH. All authors have read and agreed to the published version of the manuscript.

## Funding

This research was funded by National Natural Science Foundation of China (32071895), Fundamental Research Funds for the Central Universities, China (K20210123), and Fundamental Research Funds for the Central Universities (226-2022-00217).

## Acknowledgments

We are grateful for the rice cultivation assistance from the Zhejiang Academy of Agricultural Science.

## Conflict of interest

The authors declare that the research was conducted in the absence of any commercial or financial relationships that could be construed as a potential conflict of interest.

## Publisher’s note

All claims expressed in this article are solely those of the authors and do not necessarily represent those of their affiliated organizations, or those of the publisher, the editors and the reviewers. Any product that may be evaluated in this article, or claim that may be made by its manufacturer, is not guaranteed or endorsed by the publisher.

## Supplementary material

Supplementary Figure 1 The correlation among the hyperspectral bands.

Supplementary Figure 2 The information of the UAV platform and the field.

Supplementary Table 1 The common spectral indices and the calculation.

Supplementary Table 2 Information of the experimental samples.

Supplementary Table 3 Values of color features with different disease severity.

Supplementary Table 4 The calibration results of ATT-LSTM and SVM models by using spectral data, color features, and data fusion.

Supplementary Table 5 The results of disease severity evaluation models established using the selected important wavelengths by different feature selection methods.

Supplementary Table 6 The results of disease severity evaluation models established by fusing the selected spectra and color features.

Supplementary Table 7 The confusion matrix of correlation coefficient between disease severity and color features.

Supplementary Table 8 Information of the data acquired by UAV platform.
