# Supplementary material for: Evaluation of rice bacterial blight severity from lab to field with hyperspectral imaging technique
Source: Front Plant Sci. 2022 Oct 19;13:1037774. doi: 10.3389/fpls.2022.1037774 (PMC9627309; doi:10.3389/fpls.2022.1037774)
Supplement: Supplementary Figure 1 — The correlation among the hyperspectral bands. [file DataSheet_1.docx]

Supplementary Figures


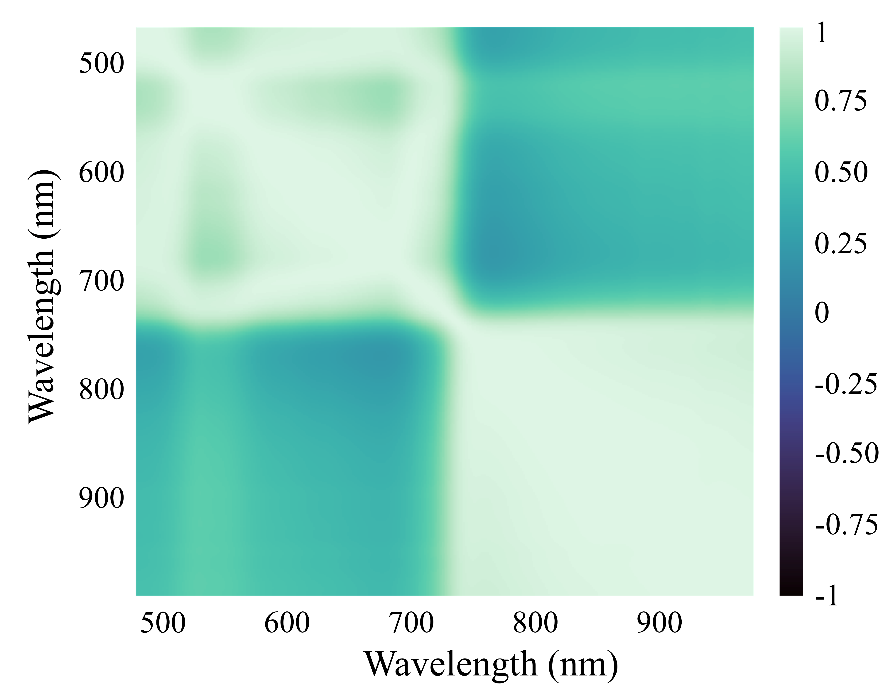


**Supplementary Figure 1.** The correlation among the hyperspectral bands.


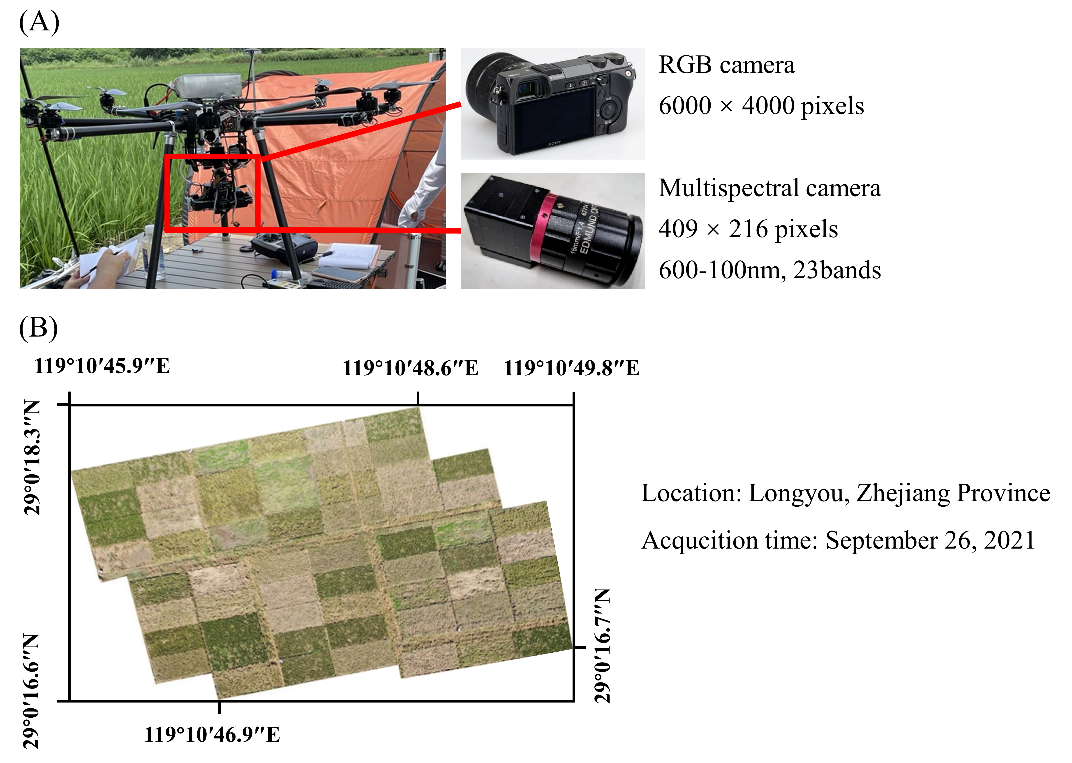


**Supplementary Figure 2.** The information of the UAV platform (A) and the field (B).
